# A cross-sectional survey of hard ticks and molecular characterization of *Rhipicephalus microplus* parasitizing domestic animals of Khyber Pakhtunkhwa, Pakistan

**DOI:** 10.1371/journal.pone.0255138

**Published:** 2021-08-05

**Authors:** Muhammad Rooman, Yasir Assad, Sadia Tabassum, Samia Sultan, Sultan Ayaz, Muhammad Fiaz Khan, Shahid Niaz Khan, Rehman Ali

**Affiliations:** 1 Department of Zoology, Hazara University Mansehra, Khyber Pakhtunkhwa, Pakistan; 2 Department of Zoology, Abdul Wali Khan University Garden Campus Mardan, Khyber Pakhtunkhwa, Pakistan; 3 College of Veterinary Sciences and Animal Husbandry, Abdul Wali Khan University Garden Campus Mardan, Khyber Pakhtunkhwa, Pakistan; 4 Department of Zoology, Faculty of Biological Sciences, Kohat University of Science and Technology, Khyber Pakhtunkhwa, Pakistan; University of Bari, ITALY

## Abstract

**Background:**

In tropical and subtropical countries, tick infestation causes major public health problems and considerable financial losses to the livestock industry. This study was aimed to assess the species composition of richness and analyze the phylogeny of *Rhipicephalus microplus* in the District Bannu of Khyber Pakhtunkhwa, Pakistan.

**Methods:**

Collected ticks were identified morphologically and DNA extracted from *R*. *microplus* was amplified and subjected to sequencing.

**Results:**

A total of 3,600 animals were examined among them 1,494 animals were found to be infested with ticks, including 669 cows, 476 buffaloes, 163 goats, and 186 sheep (*p* = 0.001). Tick infestation was significantly high (43.58%) in animals of age group (<1 year) (*p-*value = 0.027). Female animals were more (44.05%) infested with ticks than males (34.43%) (*p* = 0.001). The intensity of infestation was significantly higher in summer (77.49%) (*p* = 0.001). A total of 5,557 ticks were collected comprising three genera and six species. *R*. *microplus* was predominantly prevalent (n = 1,474; 26.52%), followed by *Rhipicephalus annulatus (*n = 1,215; 21.86%), *Hyalomma anatolicum* (n = 1,139; 20.49%), *Hyalomma marginatum* (n = 1,086; 19.54%), and *Rhipicephalus turanicus* (n = 761; 13.69%), while the least common was *Haemaphysalis aciculifer* (n = 80; 1.43%) (*p* = 0.001). Morphologically identified *R*. *microplus* species were also analyzed genetically by using two genetic markers 16S ribosomal RNA (*16S rRNA*) and internal transcribed spacer 2 (*ITS2*) genes. The phylogenetic study revealed that *R*. *microplus* is genetically diversified and clustered in clade B with *R*. *microplus* species from China, India, and Pakistan.

**Conclusion:**

Ticks infestation was significantly correlated with various factors including age, sex, season, and animal type. *R*. *microplus* genetically resembled species reported from India and China. However, major knowledge gaps concerning various species of ticks exist and many areas are still unexplored in Pakistan. Therefore, it is necessary to explore the epidemiological and molecular aspects of various tick species in other regions of southern Khyber Pakhtunkhwa.

## Introduction

Ticks are the blood-sucking arthropod parasites and a major source of emerging economic and public health concern in the regions of the tropics and subtropics [1]. Ticks act as a reservoir for several contagious pathogens of medical and veterinary importance and can transmit a wide variety of bacteria, protozoans, spirochetes, and arboviruses more than any other blood-sucking parasites [2]. Approximately, 10% of all tick species are known to transmit various pathogens [3, 4] while 80% of the cattle population is affected by ticks and tick-borne diseases [4, 5]. Besides, the transmission of infectious diseases, the parasites can also cause great loss in milk, meat production, and harm to the skin as well as hide quality [6]. The economic impact on the livestock industry due to the common cattle tick *Rhipicephalus microplus* in different regions was estimated to be 22–30 billion US$ annually [4, 5].

Various environmental factors affect the prevalence and adaptation of ticks in different regions of the world [7]. Pakistan is an agricultural country and the livestock sector is an essential part of the national budget as the 2^nd^ larger sector, which contributes 21.2% to the GDP (Gross Domestic Product) by paying 45.0% of the employees. In Pakistan, 70.0% of the population lives in rural areas, where most of them depend on livestock as the main source of income and food to survive. The population of cattle (*Bos indicus* and *Bos taurus*) 41.2 million, buffaloes (*Bubalus bubalis*) 35.6 million, goats (*Capra hircus*) 68.4 million, and 29.4 million sheep (*Ovis aries*) was estimated (based on Livestock Census 1996 & 2006) [8]. The climatic conditions of Pakistan are tremendously satisfying for the growth and survival of different tick species and other animal disease-causing parasites [9–14]. Ticks harm livestock holders, especially low-income farmers and the majority of them are unaware of different tick species and consider all ticks as a single species. The farmers also do not have any knowledge about the role of ticks in the transmission of various pathogens to human [5, 8, 15].

To date, several studies have reported the prevalence of different tick species in different provinces of Pakistan, for instance, Punjab [8, 16–18], Sindh [19], Baluchistan [20], and Khyber Pakhtunkhwa [21–23]. However, only a limited number of studies described ticks on the species level in Pakistan [8, 24, 25]. Major knowledge gaps concerning various species of ticks still exist and many areas are still unexplored in Pakistan. Therefore, this study aimed to evaluate the presence of ticks in the District Bannu of Khyber Pakhtunkhwa, Pakistan.

## Materials and method

### Study area

**Fig 1:**
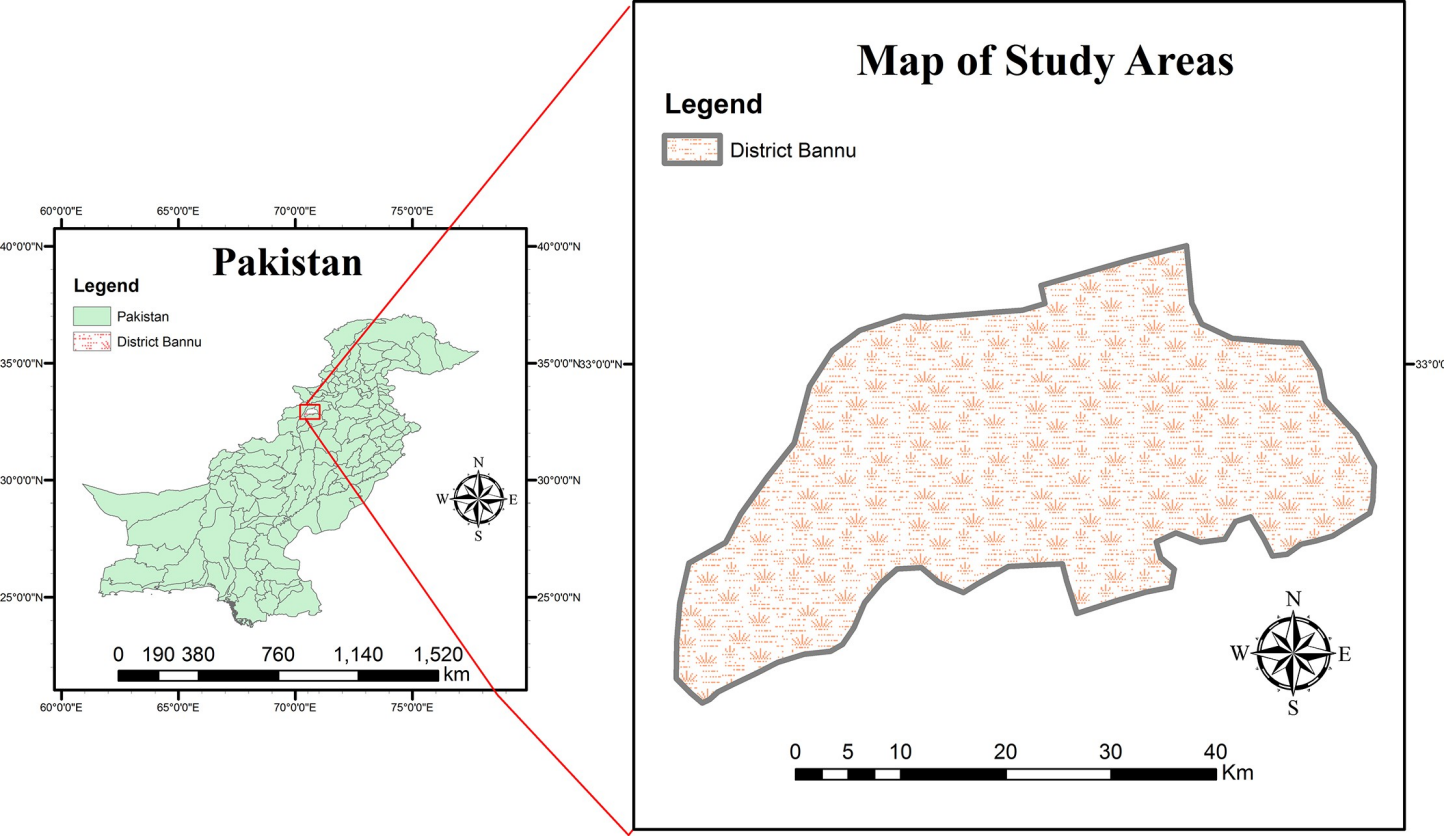
Map of the study area showing District Bannu.

The present study was carried out in District Bannu located in the southern belt of Khyber Pakhtunkhwa, Pakistan. Its borders are attached with North Waziristan to the northwest, Karak to the northeast, Lakki Marwat to the southeast, and South Waziristan to the southwest. The total region of the district is 1,227 km^2^ and out of the total, 74,196 hectares area is under cultivation (**Fig 1)**. In summer, the temperature range is about 48°C, while in winter it remains about 6°C.

### Ethical approval and consent to participation

The study was approved by the Ethical Committee of Hazara University Mansehra, Khyber Pakhtunkhwa, Pakistan. A brief purpose and aim of the study were explained and written informed consent was taken from the owner of animals before data collection.

### Sampling and morpho-taxonomic identification of ticks

The cross-sectional survey was carried out from September 2018 to August 2019. Tick specimens were collected from domestic animals (e.g. cows, buffaloes, goats, and sheep) by performing regular visits to the grazing fields and farms of the study area. The detailed history of sex, age, place, date, type of animal was recorded on a prescribed proforma. Different body parts (e.g. shoulders, dewlap, belly, head, ears, neck, back, legs, perineum, and tail) of animals were examined and forceps were used to pluck/separate ticks from the animal’s skin safely. Ticks after collection were stored in small plastic bottles to ensure safe transportation to the Parasitology Laboratory, Department of Zoology, Hazara University of Mansehra for morphological identification. The collected specimens were washed gently with phosphate buffer saline (PBS) and preserved in a separate container containing 70% ethanol. Adult ticks (both male and female) were morphologically identified with the help of a Stereomicroscope (100–200-fold magnification) and standard taxonomic keys [26–29]. The identified specimens of *R*. *microplus* were transported to the College of Veterinary Sciences and Animal Husbandry, Abdul Wali Khan University Garden Campus Mardan (AWKUM), for further molecular analysis.

### DNA extraction

Individual ticks were washed gently with ethanol and then snap-frozen in liquid nitrogen. The frozen specimens were cut into small pieces using a scalpel and ground with Genogrinder (SPEX Sample Prep). The ground tissues were placed in a sterile Eppendorf tube containing PBS for future use. Genomic DNA was extracted by using a Gene Jet Genomic DNA purification kit (Thermo Fisher Scientific) according to the standard protocol as recommended by the manufacturer. The Nanodrop ND-100 (Thermo Fisher Scientific) was used to quantify the concentration of DNA samples and placed at –20°C for further processing.

### Polymerase Chain Reaction (PCR)

Morphologically identified *R*. *microplus* species were further subjected to a polymerase chain reaction (PCR). To confirm the identity consigned to *R*. *microplus*, two genes i.e. *16S rRNA* and *ITS2* were analyzed. For the *16S rRNA* gene, a 450 base pair (bp) fragment was amplified by PCR in a thermocycler (HT, ILF, UK) using the forward primer *16S rRNA*-F (5′-AATTGCTGTAGTATTTTGAC-3′) and reverse primer *16S rRNA*-R (5′CTCCGCCTGAAGGGTCAAA-3′) as described by [30]. In the case of *ITS2*, a 750 bp fragment was amplified using primers *ITS2*-F (5′CGGATCACATATCAAGAGAG-3′) and *ITS2*-R (5′-CCCAACTGGAGTGGCCCAGTTT-3′) [31]. The total volume of the PCR reaction was 25 μL, comprising of 12.5 μL master mix [2x] (Thermo Fisher Scientific), 1.5 μL of each forward and reverse primers, 4 μL DNA template, and then 5.5 μL nuclease-free water was added to complete the final reaction. The general PCR conditions for *16S rRNA/ITS2* were followed as; an initial denaturation step at 94°C for 5 min; followed by 25 cycles of 94°C for 30 s, annealing temperature 54°C for *16S rRNA* and 57°C for *ITS2* for 30 s, initial extension temperature at 72°C for 90 s; and a final extension step at 72°C for 10 min. For validation, a negative control (distilled water) was added in each amplification reaction. The PCR products were confirmed through 2% agarose gel with ethidium bromide. DL 2,000 DNA marker (Takara) was used to find the length and concentration of the amplified products and DNA was visualized using the GeneDoc (UVP BioDoc-It Imaging System) **(S1 and S2 Figs)**. The amplicons were sent to Macrogen Inc. (Seoul, South Korea) for purification and sequencing. The obtained sequences were trimmed and edited using BioEdit (V. 7.0.) [32]. Consensus sequences were BLAST in NCBI and sequences of *R*. *microplus* and related species were retrieved from the gene bank for the downstream construction of the phylogenetic tree.

### Phylogenetic analysis

The trimmed sequences were aligned using ClustalW and Maximum-likelihood (ML) algorithm was employed to construct the phylogenetic tree in MEGA-X with bootstrapping at 1000 replicates [33]. The evolutionary distances were computed using the Tamura-Nei method [34] and are in the units of the number of base substitutions per site. Gaps and missing data were eliminated by using the partial deletion option. *Hyalomma detritum* (KC203349) was used as an outgroup for the *16S rRNA* gene, while *Ixodes scapularis* (GU319067) for the *ITS2* gene phylogenetic tree construction.

### Data analysis

The epidemiological data of different variables were analyzed using the statistical tool IBM SPSS Statistics (version 23). To determine the association between ticks and several risk factors (e.g. age, season, animal type, and sex) Chi-square Pearson’s test (*x*^2^) was used. The *p-*value (0.05) was considered to be statistically significant.

## Results

A total of 3,600 animals were studied randomly including cows, buffaloes, sheep, and goats (n = 900 each). The overall prevalence of infested animals was 41.5%. Among the studied animals, the infestation rate was higher in cows (74.3%) followed by buffalos (53.0%), sheep (21.0%), and goats (18.1%) **(Table 1).** This rate of infestation was highly significant among the studied animals (P<0.001).

**Table 1:** Overall prevalence of hard ticks in District Bannu.

Animal host	Infested/ Animals examined (%)	Pearson’s Chi-square test *(x*^*2*^*)*	*p*-value
Cows Buffaloes Sheep Goats	669/900 (74.3)	811.419	0.001
	476/900 (53.0)		
	186/900 (21.0)		
	163/900 (18.1)		
**Total**	**1,494/3,600 (41.5)**		

Age-wise comparison revealed a high prevalence in calves/lams/kid (43.58%) than younger (41.0%) and adult (40.2%) animals (*p* = 0.027). Similarly, the majority of infested animals were females (44.0%) than males (34.4%). This difference was also statistically highly significant (*p* = 0.001) **(Table 2)**. Season-wise prevalence depicted highest percentage in summer (77.5%), followed by spring (65.1%), autumn (30.0%), and winter (11.4%). While month-wise the highest prevalence was recorded in July (83.2%) and the lowest prevalence (5.9%) in December **(Table 3)**.

**Table 2:** Age and sex-wise prevalence of hard ticks in District Bannu.

Variables	Cows	Buffaloes	Sheep	Goats	Prevalence (%)	Pearson’s Chi-square *(x*^*2*^*)*	*p*-value
	Infested/ Examined (%)	Infested/Examined (%)	Infested/Examined (%)	Infested/Examined (%)			
**Age (year)**							
Calf/lamb/kid (<1)	219/299 (84.0)	177/349 (65.2)	62/262 (26.5)	58/274 (24.0)	44.0	7.195	.027
Younger (2–3)	219/294 (79.0)	151/301 (47.0)	61/292 (20.0)	45/280 (17.0)	41.0		
Adult (>5)	231/307 (52.0)	148/250 (45.0)	63/346 (13.5)	60/346 (13.0)	40.2		
**Sex**							
Male	154/193 (62.0)	82/229 (36.0)	45/229 (20.0)	53/316 (17.0)	34.4	14.145	0.001
Female	515/707 (79.1)	394/671 (59.0)	141/671 (21.0)	110/584 (19.0)	44.0		

**Table 3:** Season-wise prevalence of hard ticks in District Bannu.

Variables		Cows	Buffaloes	Sheep	Goats	Overall prevalence%		Pearson’s Chi-square test *(x*^*2*^*)*	*p*-value
Seasons	Months	Infested/Examined	Infested/Examined	Infested/Examined	Infested/Examined	Month-wise %	Season-wise %		
Spring	Sep	72/83	54/88	18/41	20/41	64.82	65.1	1224.196	0.001
	Oct	43/55	39/64	10/52	12/54	46.22			
	Nov	19/59	27/74	8/79	13/86	22.48			
	Dec	7/56	12/82	4/140	2/143	5.93			
	Jan	17/60	14/91	4/141	4/141	9.00			
	Feb	29/64	21/65	1/105	7/115	16.61			
Winter	Mar	46/64	33/64	7/91	7/93	29.80	11.4		
	Apr	64/74	37/71	19/68	8/59	47.05			
	May	68/72	41/60	15/34	13/34	68.50			
	Jun	88/90	59/69	22/40	20/41	78.09			
	Jul	128/140	71/83	40/56	29/49	83.22			
	Aug	88/89	68/89	38/53	28/44	80.72			
Autumn							30.0		
Summer							77.5		

All ruminants including cows, buffalos, sheep, and goats, regardless of their geographic position were examined for hard tick species. A total of 5,557 adult ticks were collected from various body parts of infested animals. Three genera comprising of *Rhipicephalus*, *Hyalomma*, and *Haemaphysalis*, and 6 species i.e. *R*. *microplus*, *Rhipicephalus turanicus*, *Rhipicephalus annulatus*, *Hyalomma marginatum*, *Hyalomma anatolicum*, and *Haemaphysalis aciculifer* were identified morphologically. *R*. *microplus* (n = 1,474; 26.0%) was observed to be the most dominant species, followed by *R*. *annulatus* (n = 1,215; 21.0%), *Hya*. *anatolicum* (n = 1,139; 20.0%), *Hya*. *marginatum* (n = 1,086; 19.0%), and *R*. *turanicus* (n = 761; 13.0%), while the least common was *Hae*. *aciculifer* (n = 80; 1.4%) (*p* = 0.001) **(Table 4)**.

**Table 4:** Genus and species-wise prevalence of hard ticks in District Bannu.

Variables	No. of ticks	No. of infested animals	Percentage (%)	Pearson’s Chi-square test *(x*^*2*^*)*	*p*-value
**Genera**					
*Haemaphysalis*	80	20	1.4	3600.000	0.001
*Hyalomma*	2,225	587	39		
*Rhipicephalus*	3,450	887	60		
**Species**					
*Haemaphysalis aciculifer*	80	20	1.4	3600.000	0.001
*Hyalomma anatolicum*	1,139	332	20		
*Hyalomma marginatum*	1,086	255	19		
*Rhipicephalus annulatus*	1,215	309	21		
*Rhipicephalus turanicus*	761	225	13		
			
*Rhipicephalus microplus*	1,474	353	26		
**Total**	**5,755**	**1,494**	**100**		

BLAST analysis showed a remarkable (100%) identity of *16S rRNA* gene nucleotide sequences with species from India (accession no. MG811555; MF946459; KY458969), Pakistan (accession no. MN726558; MT799952), and China (accession no. KU664521). Similarly, *ITS2* nucleotide sequence also showed high resemblance (100%) with species reported from China (accession no. MK224585; MK224584; KX450289), India (accession no. MK621182; MH598985; MF946462), and Pakistan (accession no. MW580928; MW580866). The nucleotide sequences of *16S rRNA* (MZ540266, MZ540267, MZ540268) and *ITS2* (MZ542565) of the current study were deposited to the NCBI GenBank.

**Fig 2:**
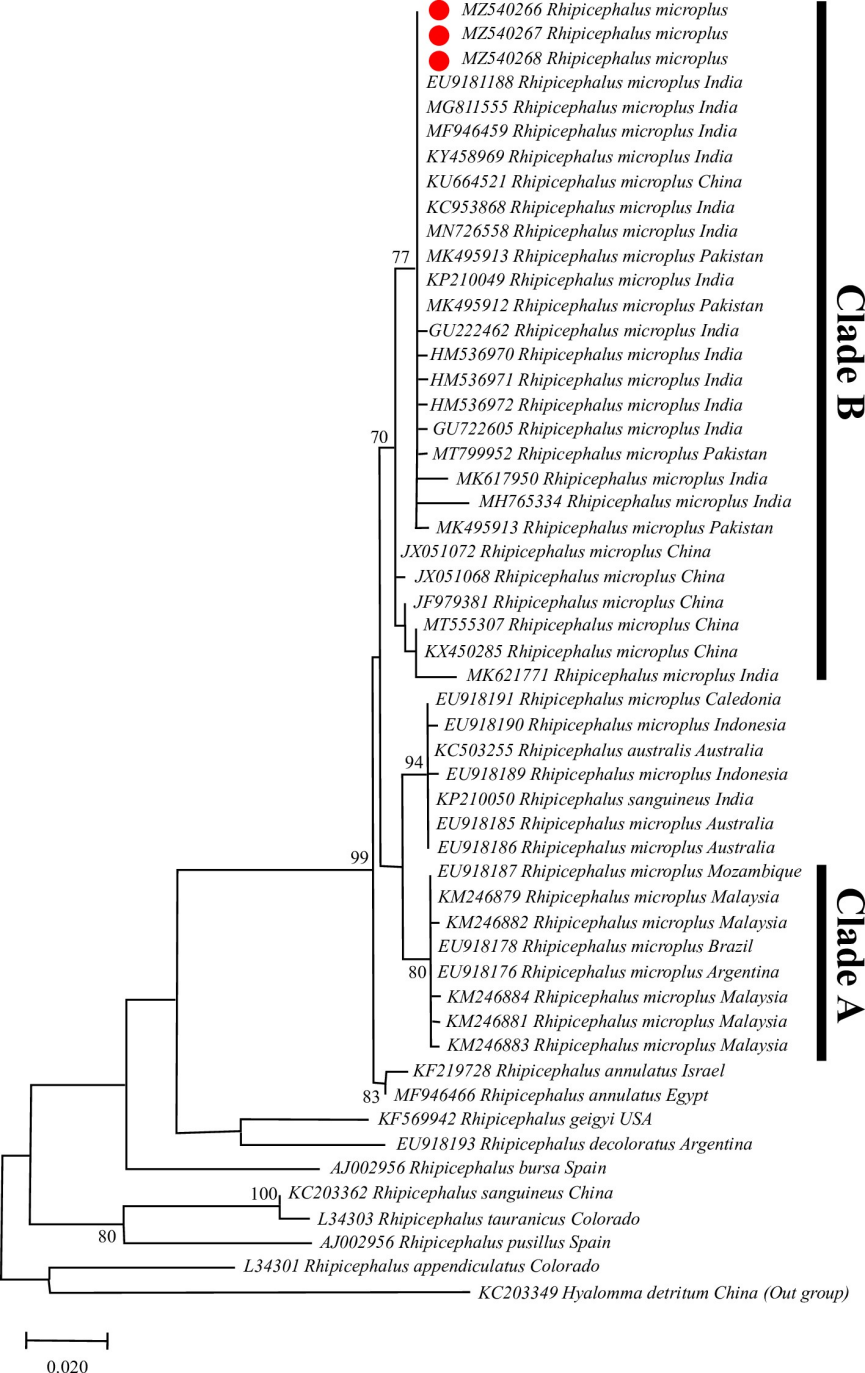
Phylogenetic tree of *16S rRNA* sequences of the genus *Rhipicephalus* and using *Hyalomma detritum* sequence as outgroup. The tree was inferred using the Maximum likelihood method and evolutionary distances were computed using the Tamura-Nei model. The bootstraps values (1000 replicates) are shown next to the taxa. The sequences of the present study are marked in red.

**Fig 3:**
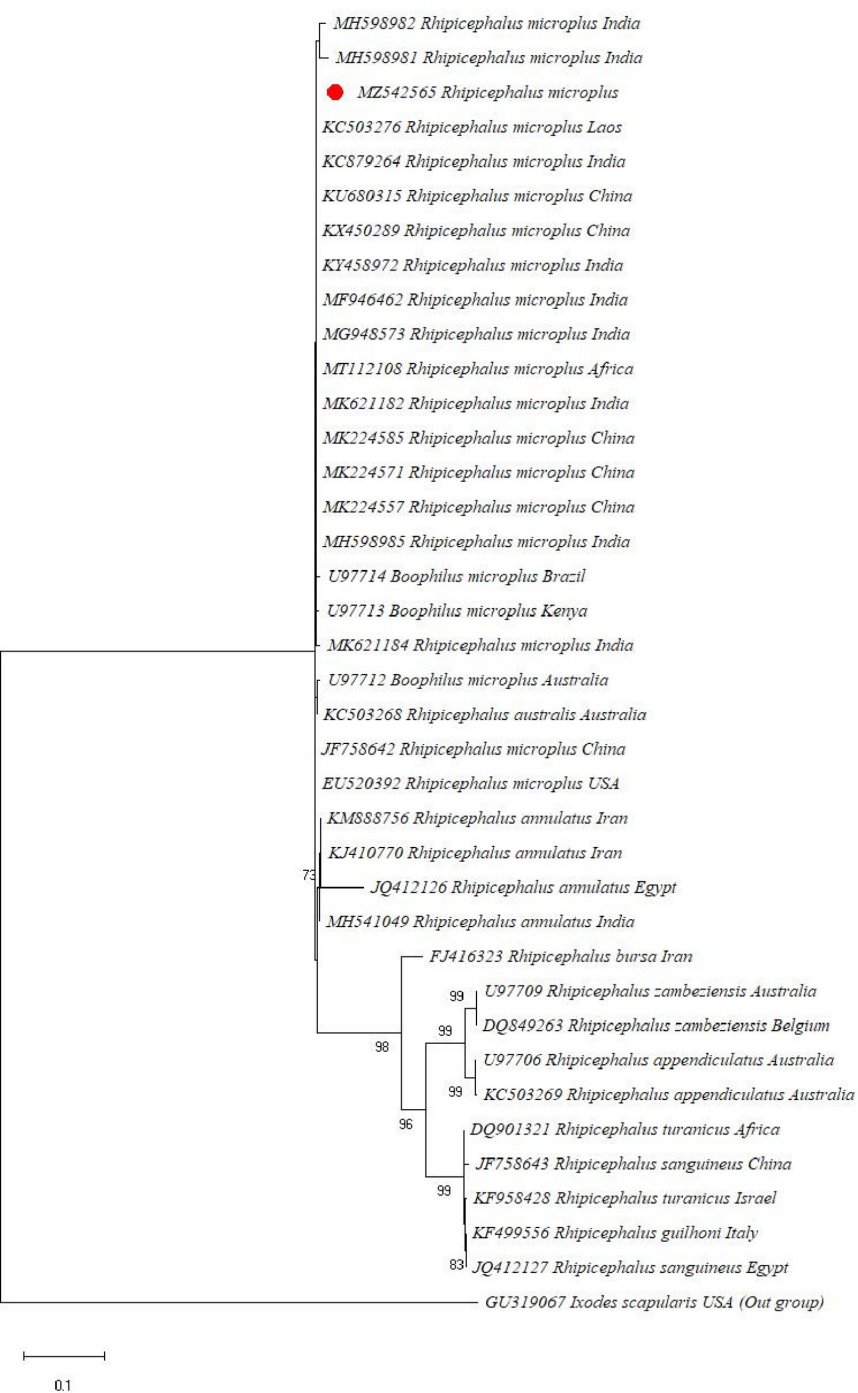
Phylogenetic tree of *ITS2* sequences of the genus *Rhipicephalus* and using *Ixodes scapularis sequence* as outgroup. The tree was inferred using the Maximum likelihood method and evolutionary distances were computed using the Tamura-Nei model. The bootstraps values (1000 replicates) are shown next to the taxa. The sequences of the present study are marked in red.

The *16S rRNA* ML phylogenetic tree has strong bootstrap support for monophyly of *R*. *microplus* and its division into clade A and clade B. The clade A was comprised of *R*. *microplus* from South Africa (Mozambique), Malaysia, and America (Argentina, Brazil). While the clade B was comprised of *R*. *microplus* from China, India, and Pakistan. *R*. *microplus* from India, China, and Pakistan collectively depicted a moderate bootstrap value (77%), and *R*. *microplus* from Malaysia, America, and Africa have shown (80%). The tree also revealed a well-supported clade (94%) of *R*. *australis* from Australia, Caledonia, and Indonesia **(Fig 2)**. On the other hand, *ITS2* analysis depicted that all species of *R*. *microplus* complex were clustered together and well-supported monophyly (**Fig 3**). However, this tree revealed a little structure within the *R*. *microplus* complex other than the average support for monophyly of *R*. *annulatus* (73%) and the two *R*. *australis* from Australia (59%). *R*. *microplus* specimens from China have a similar *ITS2* sequence to several other *R*. *microplus* specimens. The *ITS2* tree has also strong support for the placement of most other *Rhipicephalus* species and places a clade consisting of *R*. *bursa*, and sister clades of *R*. *zambeziensis*, *R*. *appedniculatus*, *R*. *turanicus*, *R*. *sanguineus*, and *R*. *guilhoni*.

## Discussion

In the current cross-sectional study, the overall prevalence of ticks was reported to be 41.47%. In different regions of Pakistan, other investigators [16, 20, 21] also reported the prevalence of tick infestation where the percentage was 75.0, 11.3, 13.4, and 35.0%, respectively. The difference in the current and previous studies might be due to differences in species, breed, sex, age, host [35, 36], sample size, sample period, different management systems, climatic conditions, humidity, and environmental conditions [37]. Higher tick prevalence can also be justified by a systematic rainy season that makes the environment suitable for tick propagation [38]. Among animals, the highest prevalence was reported in cows (74.3%) and buffalos (52.7%). This study was consistent with the study conducted by [17] where the overall prevalence was 36.5%, while cows and buffaloes were highly infested at 37.5 and 42.4%, respectively. In large ruminants, the higher prevalence of ticks infestation in the cow population may be due to the thinner skin of cows as compared to buffaloes [39]. Moreover, the cows adapt to a dried habitat, while buffaloes prefer marshy places [40]. The reason for the higher prevalence in small ruminants may not be evident however, genetics may play a vital role [41].

Females were more affected in the current study which coincided with the previous study conducted in ruminants [42, 43]. In Pakistani society, male animals are mostly used for breeding purposes throughout the year. Moreover, in Muslim countries male animals are sacrificed at “Eid-ul-Adha” (holy festival of Muslims) therefore, they take more consideration as compared to female animals. Hence, the male would have a low ticks burden and physically groom well. Besides, pregnancy and lactation period may also be one of the factors that decrease the resistance in females [44].

Tick infestation varied throughout the year and the highest was recorded during June, July, and August. Similar results were reported by [18, 45] from Punjab, where the infestation rate was higher in June and July. It was observed that infestation was higher in the summer season because in summer the weather becomes warm, humid, and makes the environment suitable for tick growth and multiplication. These results were following the previous studies [46–48]. Various ecological factors like different study areas, climatic conditions, rainfall, temperature, and humidity may also affect tick infestation rates [37]. Some other factors like farming practices [49], host availability, and nutrition status might have a vital role [36]. Similarly, Mondal et al. (2011) also reported a higher prevalence in the summer season (41.6%) followed by winter (31.5%) [50].

The age of the animal has an important role in the prevalence of tick infestation [21]. It was observed, that calves were more susceptible to tick infestation than other age group animals. It might be since adults are productive animals, therefore, adults are carefully looked after to increase production. Besides, adult animals may also acquire resistance to tick infestation due to repeated exposure over time [51]. Similar trends of ticks infestation were observed in the past by other investigators [21, 52].

In our finding, it was observed that the genus *Rhipicephalus* was more prevalent 62.0% than other tick genera. This finding was in accordance with previous studies [22, 53]. The higher infestation rate of the predominant genera might be due to the rainfall ranging from high to temperate [53]. The decline configuration of the genus *Hyalomma* was due to the rainy to semi-arid features of temporal areas, as stated by [53, 54]. Similarly, at the species level, the *R*. *microplus* was the most prevalent species in our study which was present in all infected animals throughout the study area. These results were in parity with the study carried out by [22] where the author observed *R*. *microplus* in a massive number. Retaining water capability beneath the layer of the soil and increase in humidity of the study area sponsors the distribution and propagation of this tick species. In the near past, it was discovered that *R*. *microplus* is a complex, comprising of five taxa: *R*. *microplus* clade A, *R*. *microplus* clade B, and *R*. *microplus* clade C, *R*. *australis*, and *R*. *annulatus* [55, 56]. Two *Hyalomma* species (*Hya*. *anatolicum and Hya*. *marginatum*) and one *Haemaphysalis* species (*Hae*. *aciculifer*) were also reported in the present study which was previously documented from Pakistan [22]. The *Hyalomma* species act as a vector and transmit various diseases like *Theileria annulata* and *Theileria lestoquardi* in Pakistan [57]. Also, *Hyalomma* species have long mouthparts that can damage the cattle hides extremely and mostly target the teat of the cattle that may cause difficulty in calves suckling [58]. *Heamaphysalis* species also transmit various diseases like theileriosis and babesiosis in sheep and goats [46]. Furthermore, the causative agents of bovine anaplasmosis in Pakistan are mainly transmitted by *R*. *microplus* and *Hyalomma* species [59]. By comparing the studies in Pakistan to other countries the data on tick epidemiology and genetic diversity is limited and insufficient. However, the tick infestation rate was almost same among Khyber Pakhtunkhwa and other provinces of the country [42, 47]. It may be due to the free movement of animals across the province, districts, and keeping of mixed species in the same area, particularly in Khyber Pakhtunkhwa [22].

The genetic markers i.e. *16S rRNA* and *ITS2* define the intraspecific genetic diversity and phylogeographical connections of the important global pest of livestock, *R*. *microplus*. Previously, both genetic markers have been used to investigate the precise relationship among hard ticks such as *R*. *microplus* complex [25, 55, 56, 60, 61]. In phylogenetic analysis *ITS2* gene has shown to be a useful genetic marker however, it contains little intraspecific variation but significant interspecific inconsistency [62]. On the other hand, *16S rRNA* has been also used for the investigation of the phylogenetic relationships of various important tick species across the globe [63–66]. For good identification of the cryptic species of *R*. *microplus* in different geographical regions, *16S rRNA* sequences were used [30]. Phylogenetic analysis of the *16S rRNA* gene revealed two different genetic clades of *R*. *microplus*. Similar studies were also reported elsewhere, where two genetic clades were confirmed [8, 25, 31, 56, 60, 67]. However, the genetic marker *ITS2* was clustered together in a single clade on the ML phylogenetic tree. It means that this marker cannot differentiate within the same species and support the monophyly of the *R*. *microplus* complex. This finding was similar to a previous study reported from Pakistan [25]. To indicate the evolutionary connections of *R*. *microplus*, the *16S rRNA* gene provides sufficient power as compared to the *ITS2* because mitochondrial DNA sequences evolve rapidly and are inherited maternally [66, 68]. From the current findings, it is clear that the *16S rRNA* marker has a well-resolving power as compared to the ITS2 gene.

## Conclusion

This is the first attempt to explore the prevalence of hard tick fauna as well as molecular characterization of *R*. *microplus* in the Bannu district. We reported three genera and six species i.e. *R*. *microplus*, *R*. *turanicus*, *R*. *annulatus*, *Hya*. *marginatum*, *Hya*. *anatolicum*, and *Hae*. *aciculifer*, where *R*. *microplus* was reported as one of the most prevalent species. Various factors like age, sex, season and animal type significantly affected the tick infestation rate. It was also concluded that genetically *R*. *microplus* showed more similarity with that of India and China. However, major knowledge gaps concerning various species of ticks exist and many areas are still unexplored in Pakistan. Therefore, it is necessary to explore the epidemiological and molecular aspects of various tick species in other regions of southern Khyber Pakhtunkhwa. This study will be useful in the investigation and designing control strategies for ticks control in Pakistan.

## Supporting information

S1 Fig *16S rRNA* amplified product. A 2000 ladder was used. 1–4 represent samples of the present study. N represents negative control and P represents the positive control.
(DOCX)

S2 Fig *ITS2* amplified product. A 2000 ladder was used. 1–3 represent samples of the present study. N represents negative control and P represents the positive control.
(DOCX)

S1 Raw images (PDF)

## References

[1] de La Fuente J, Antunes S, Bonnet S, Cabezas-Cruz A, Domingos AG, Estrada-Peña A, et al. Tick-pathogen interactions and vector competence: identification of molecular drivers for tick-borne diseases. Front Cell Infect Microbiol. 2017;7:114. doi: 10.3389/fcimb.2017.00114 28439499 PMC5383669

[2] Sherrard-Smith E, Chadwick E, Cable J. Abiotic and biotic factors associated with tick population dynamics on a mammalian host: *Ixodes hexagonus* infesting otters, Lutra lutra. PLoS One. 2012;7(10):e47131. doi: 10.1371/journal.pone.0047131 23071736 PMC3465257

[3] Parola P, Raoult D. Ticks and tickborne bacterial diseases in humans: an emerging infectious threat. Clin Infect Dis. 2001;32(6):897–928. doi: 10.1086/319347 11247714

[4] Lew-Tabor AE, Rodriguez Valle M. Erratum to A review of reverse vaccinology approaches for the development of vaccines against ticks and tick borne diseases. Ticks Tick Borne Dis. 2016;7(6):1236–7. doi: 10.1016/j.ttbdis.2016.07.008 26723274

[5] Jabbar A, Abbas T, Saddiqi HA, Qamar MF, Gasser RB. Tick-borne diseases of bovines in Pakistan: major scope for future research and improved control. Parasit vectors. 2015;8(1):283. doi: 10.1186/s13071-015-0894-2 25994588 PMC4443554

[6] Grisi L, Leite RC, Martins JR, Barros AT, Andreotti R, Cancado PH, et al. Reassessment of the potential economic impact of cattle parasites in Brazil. Rev Bras Parasitol Vet. 2014;23(2):150–6. doi: 10.1590/s1984-29612014042 25054492

[7] Estrada-Peña A. Climate, niche, ticks, and models: what they are and how we should interpret them. Parasitol Res. 2008;103(1):87–95. doi: 10.1007/s00436-008-0932-5 19030890

[8] Rehman A, Nijhof AM, Sauter-Louis C, Schauer B, Staubach C, Conraths FJ. Distribution of ticks infesting ruminants and risk factors associated with high tick prevalence in livestock farms in the semi-arid and arid agro-ecological zones of Pakistan. Parasit vectors. 2017;10(1):190. doi: 10.1186/s13071-017-2138-0 28420420 PMC5395890

[9] Ali R, Rooman M, Mussarat S, Norin S, Ali S, Adnan M, et al. A systematic review on comparative analysis, toxicology, and pharmacology of medicinal plants against *Haemonchus contortus*. Front Pharmacol. 2021;12:644027. doi: 10.3389/fphar.2021.644027 34040520 PMC8141741

[10] Khan SN, Ali R, Khan S, Norin S, Rooman M, Akbar NU, et al. Cystic echinococcosis: an emerging zoonosis in southern regions of Khyber Pakhtunkhwa, Pakistan. BMC Vet Res. 2021;17(1):139. doi: 10.1186/s12917-021-02830-z 33794898 PMC8015088

[11] Ali R, Khan S, Khan M, Adnan M, Ali I, Khan TA, et al. A systematic review of medicinal plants used against *Echinococcus granulosus*. PLoS One. 2020;15(10):e0240456. doi: 10.1371/journal.pone.0240456 33048959 PMC7553295

[12] Rehman ZU, Zahid O, Rashid I, Ali Q, Akbar MH, Oneeb M, et al. Genetic diversity and multiplicity of infection in *Fasciola gigantica* isolates of Pakistani livestock. Parasitol Int. 2020;76:102071. doi: 10.1016/j.parint.2020.102071 32045674

[13] Zafar A, Khan MK, Sindhu D, Abbas RZ, Masood S, Abbas Z, et al. Seroprevalence of *Fasciola hepatica* in small ruminants of District Chakwal, Punjab, Pakistan. Pak Vet J. 2019;39(1).

[14] Ali Q, Rashid I, Shabbir MZ, Akbar H, Shahzad K, Ashraf K, et al. First genetic evidence for the presence of the rumen fluke *Paramphistomum epiclitum* in Pakistan. Parasitol Int. 2018;67(5):533–7. doi: 10.1016/j.parint.2018.05.005 29758277

[15] Karim S, Budachetri K, Mukherjee N, Williams J, Kausar A, Hassan MJ, et al. A study of ticks and tick-borne livestock pathogens in Pakistan. PLoS Negl Trop Dis. 2017;11(6):e0005681. doi: 10.1371/journal.pntd.0005681 28650978 PMC5501686

[16] Ahmad Z, Anwar Z, Adnan M, Imtiaz N, Rashid HU, Gohar F. Collection and prevalence of ticks in cattles and buffaloes from free-range management systems of Islamabad. J Basic Appl Zool. 2019;80(1):12.

[17] Batool M, Nasir S, Rafique A, Yousaf I, Yousaf M. Prevalence of Tick Infestation in Farm Animals from Punjab, Pakistan. Pak Vet J. 2019;39(3).

[18] Atif FA, Khan MS, Iqbal HJ, Arshad GM, Ashraf E, Ullah S. Prevalence of *Anaplasma marginale*, *Babesia bigemina* and *Theileria annulata* infections among cattle in Sargodha District, Pakistan. Afr J Agric Res. 2012;7(22):302–3307.

[19] Soomro MH, Soomro SP, Bhutto MB, Akbar Z, Yaqoob M, Arijo AG. Prevalence of ticks in buffaloes in the upper Sindh Pakistan. Buffalo Bull. 2014;33(3):323–7.

[20] Kakar M, Khan M, Khan M, Ashraf K, Kakar M, Jan S, et al. Prevalence of tick infestation in different breeds of cattle in Balochistan. J, Ani Plant Sci. 2017;27(3).

[21] Manan A, Khan Z, Ahmad B, editors. Prevalence and identification of ixodid tick genera in frontier region Peshawar. J Agri Biol Sci; 2007: Citeseer.

[22] Farooqi SH, Ijaz M, Saleem MH, Rashid MI, Oneeb M, Khan A, et al. Distribution of ixodid tick species and associated risk factors in temporal zones of Khyber Pakhtunkhwa Province, Pakistan. Pak J Zool. 2017;49(6).

[23] Sajid MS, Iqbal Z, Shamim A, Siddique RM, HASSAN MJU, Rizwan HM. Distribution and abundance of ticks infesting livestock population along Karakorum highway from Mansehra to Gilgit, Pakistan. J Hellenic Vet Med Soc. 2017;68(1):51–8.

[24] Zeb J, Szekeres S, Takács N, Kontschán J, Shams S, Ayaz S, et al. Genetic diversity, piroplasms and trypanosomes in *Rhipicephalus microplus* and *Hyalomma anatolicum* collected from cattle in northern Pakistan. Exp Appl Acarol. 2019;79(2):233–43. doi: 10.1007/s10493-019-00418-9 31578647

[25] Ali A, Khan MA, Zahid H, Yaseen PM, Khan MQ, Nawab J, et al. Seasonal dynamics, record of ticks infesting humans, wild and domestic animals and molecular phylogeny of *Rhipicephalus microplus* in Khyber Pakhtunkhwa Pakistan. Front Physiol. 2019;10. doi: 10.3389/fphys.2019.00010 31379587 PMC6646419

[26] Walker AR. Ticks of domestic animals in Africa: a guide to identification of species: Bioscience Reports Edinburgh; 2003.

[27] Walker JB, Keirans JE, Horak IG. The genus Rhipicephalus (Acari, Ixodidae): a guide to the brown ticks of the world: Cambridge University Press; 2005.

[28] Geevarghese G, Dhanda V. The Indian Hyalomma ticks (Ixodoidea: Ixodidae). The Indian Hyalomma ticks (Ixodoidea: Ixodidae). 1987.

[29] Geevarghese G, Mishra A. Haemaphysalis ticks of India: Elsevier; 2011.

[30] Brahma RK, Dixit V, Sangwan AK, Doley R. Identification and characterization of *Rhipicephalus* (*Boophilus*) *microplus* and *Haemaphysalis bispinosa* ticks (Acari: Ixodidae) of North East India by ITS2 and 16S rDNA sequences and morphological analysis. Exp Appl Acarol. 2014;62(2):253–65. doi: 10.1007/s10493-013-9732-4 23990074

[31] Csordas BG, Garcia MV, Cunha RC, Giachetto PF, Blecha IMZ, Andreotti R. New insights from molecular characterization of the tick *Rhipicephalus* (*Boophilus*) *microplus* in Brazil. Rev Bras Parasitol Vet. 2016;25(3):317–26. doi: 10.1590/S1984-29612016053 27579530

[32] Hall TA, editor BioEdit: a user-friendly biological sequence alignment editor and analysis program for Windows 95/98/NT. Nucleic Acids Symp Ser; 1999: [London]: Information Retrieval Ltd., c1979–c2000.

[33] Tamura K, Nei M, Kumar S. Prospects for inferring very large phylogenies by using the neighbor-joining method. Proceedings of the National Academy of Sciences. 2004;101(30):11030–5. doi: 10.1073/pnas.0404206101 15258291 PMC491989

[34] Kimura M. A simple method for estimating evolutionary rates of base substitutions through comparative studies of nucleotide sequences. J Mol Evol. 1980;16(2):111–20. doi: 10.1007/BF01731581 7463489

[35] Swai E, Mbise A, Kessy V, Kaaya E, Sanka P, Loomu P. Farm constraints, cattle disease perception and tick management practices in pastoral Maasai community-Ngorongoro, Tanzania. Livest Res Rural. 2005;17(2).

[36] Yacob H, Ataklty H, Kumsa B. Major ectoparasites of cattle in and around Mekelle, northern Ethiopia. Entomol Res. 2008;38(2):126–30.

[37] Greenfield B. Environmental parameters affecting tick (*Ixodes ricinus*) distribution during the summer season in Richmond Park, London. Bioscience Horizons. 2011;4(2):140–8.

[38] Lima W, Ribeiro M, Guimaraes M. Seasonal variation of *Boophilus microplus* (Canestrini, 1887)(Acari: Ixodidae) in cattle in Minas Gerais St{Lima, 2000 #29}ate, Brazil. Trop Anim Health Prod. 2000;32(6):375–80. doi: 10.1023/a:1005229602422 11147277

[39] Abbasi F, Abbasi IHR, Nissa TF, Bhutto ZA, Arain MA, Soomro RN, et al. Epidemiological study of tick infestation in buffalo of various regions of district Khairpur, Pakistan. Vet World. 2017;10(6):688–94. doi: 10.14202/vetworld.2017.688-694 28717323 PMC5499088

[40] Sajid MS, Iqbal Z, Khan MN, Muhammad G. In vitro and in vivo efficacies of ivermectin and cypermethrin against the cattle tick *Hyalomma anatolicum anatolicum* (Acari: Ixodidae). Parasitol Res. 2009;105(4):1133–8. doi: 10.1007/s00436-009-1538-2 19562374

[41] Jonsson NN, Piper EK, Constantinoiu CC. Host resistance in cattle to infestation with the cattle tick *Rhipicephalus microplus*. Parasite Immunol. 2014;36(11):553–9. doi: 10.1111/pim.12140 25313455

[42] Iqbal A, Sajid MS, Khan MN, Khan MK. Frequency distribution of hard ticks (Acari: Ixodidae) infesting bubaline population of district Toba Tek Singh, Punjab, Pakistan. Parasitol Res. 2013;112(2):535–41. doi: 10.1007/s00436-012-3164-7 23086441

[43] Asmaa NM, ElBably MA, Shokier KA. Studies on prevalence, risk indicators and control options for tick infestation in ruminants. Beni-suef Uni J Basic Appl Sci. 2014;3(1):68–73.

[44] Sutherst R, Maywald G, Kerr J, Stegeman D. The effect of cattle tick (*Boophilus microplus*) on the growth of *Bos indicus*× *B. taurus* steers. Aust J Agric Res. 1983;34(3):317–27.

[45] Mustafa I, Shabbir RMK, Subhani M, Ahmad I, Aleem R, Jamil S, et al. Seasonal activity of tick infestation in goats and buffalo of Punjab province (district Sargodha), Pakistan. Kafkas Univ Vet Fak Derg. 2014;20:655–62.

[46] Ghosh S, Bansal GC, Gupta SC, Ray D, Khan MQ, Irshad H, et al. Status of tick distribution in Bangladesh, India and Pakistan. Parasitol Res. 2007;101 Suppl 2:S207–16. doi: 10.1007/s00436-007-0684-7 17823830

[47] Durrani AZ, Kamal N. Identification of ticks and detection of blood protozoa in friesian cattle by polmerase chain reacton test and estimation of blood parameters in district Kasur, Pakistan. Trop Anim Health Prod. 2008;40(6):441–7. doi: 10.1007/s11250-007-9117-y 18575972

[48] Rony S, Mondal M, Begum N, Islam M, Affroze S. Epidemiology of ectoparasitic infestations in cattle at Bhawal forest area, Gazipur. Bangladesh J Vet Med. 2010;8(1):27–33.

[49] Sajid MS, Iqbal Z, Khan MN, Muhammad G, Needham G, Khan MK. Prevalence, associated determinants, and in vivo chemotherapeutic control of hard ticks (Acari: Ixodidae) infesting domestic goats (*Capra hircus*) of lower Punjab, Pakistan. Parasitol Res. 2011;108(3):601–9. 10.1007/s00436-010-2103-8 20924608

[50] Kabir M, Mondal M, Eliyas M, Mannan M, Hashem M, Debnath N, et al. An epidemiological survey on investigation of tick infestation in cattle at Chittagong District, Bangladesh. Afr J Microbiol Res. 2011;5(4):346–52.

[51] Patel G, Shanker D, Jaiswal AK, Sudan V, Verma SK. Prevalence and seasonal variation in ixodid ticks on cattle of Mathura district, Uttar Pradesh. J Parasit Dis. 2013;37(2):173–6. doi: 10.1007/s12639-012-0154-8 24431564 PMC3793110

[52] Singh NK, Rath SS. Epidemiology of ixodid ticks in cattle population of various agro-climatic zones of Punjab, India. Asian Pac J Trop Med. 2013;6(12):947–51. doi: 10.1016/S1995-7645(13)60169-8 24144025

[53] Islam MK, Alim MA, Tsuji N, Mondal MMH. An investigation into the distribution, host-preference and population density of ixodid ticks affecting domestic animals in Bangladesh. Trop Anim Health Prod. 2006;38(6):485–90. doi: 10.1007/s11250-006-4381-9 17243476

[54] Harwood R, James M. Ticks and tick-associated diseases. Entomology in Human and Animal Health. 1979:371–416.

[55] Burger TD, Shao R, Barker SC. Phylogenetic analysis of mitochondrial genome sequences indicates that the cattle tick, *Rhipicephalus* (*Boophilus*) *microplus*, contains a cryptic species. Mol Phylogenet Evol. 2014;76:241–53. doi: 10.1016/j.ympev.2014.03.017 24685498

[56] Low VL, Tay ST, Kho KL, Koh FX, Tan TK, Lim YA, et al. Molecular characterisation of the tick *Rhipicephalus microplus* in Malaysia: new insights into the cryptic diversity and distinct genetic assemblages throughout the world. Parasit vectors. 2015;8:341. doi: 10.1186/s13071-015-0956-5 26104478 PMC4482097

[57] Ali Z, Maqbool A, Muhammad K, Khan M, Younis M. Prevalence of *Theileria annulata* infected hard ticks of cattle and buffalo in Punjab, Pakistan. DNA. 2013;862:846.

[58] Tadesse F, Abadfaji G, Girma S, Jibat T. Identification of tick species and their preferred site on cattles body in and around Mizan Teferi, Southwestern Ethiopia. J Vet Med Anim Health. 2012;4(1):1–5.

[59] Ashraf QU, Khan AU, Khattak RM, Ali M, Shaikh RS, Ali M, et al. A report on the high prevalence of *Anaplasma* sp. in buffaloes from two provinces in Pakistan. Ticks Tick Borne Dis. 2013;4(5):395–8. doi: 10.1016/j.ttbdis.2013.04.001 23743023

[60] Lv J, Wu S, Zhang Y, Chen Y, Feng C, Yuan X, et al. Assessment of four DNA fragments (COI, 16S rDNA, ITS2, 12S rDNA) for species identification of the Ixodida (Acari: Ixodida). Parasit vectors. 2014;7(1):93. doi: 10.1186/1756-3305-7-93 24589289 PMC3945964

[61] Coimbra-Dores MJ, Maia-Silva M, Marques W, Oliveira AC, Rosa F, Dias D. Phylogenetic insights on Mediterranean and Afrotropical *Rhipicephalus* species (Acari: Ixodida) based on mitochondrial DNA. Exp Appl Acarol. 2018;75(1):107–28. doi: 10.1007/s10493-018-0254-y 29605833

[62] Barker SC. Distinguishing species and populations of rhipicephaline ticks with its 2 ribosomal RNA. J Parasitol. 1998;84:887–92. 9794625

[63] Black WC, Piesman J. Phylogeny of hard-and soft-tick taxa (Acari: Ixodida) based on mitochondrial 16S rDNA sequences. Proc Natl Acad Sci. 1994;91(21):10034–8. doi: 10.1073/pnas.91.21.10034 7937832 PMC44952

[64] Caporale DA, Rich SM, Spielman A, Telford SR, Kocher TD. Discriminating between Ixodes ticks by means of mitochondrial DNA sequences. Mol Phylogen Evol. 1995;4(4):361–5. doi: 10.1006/mpev.1995.1033 8747292

[65] Chao L-L, Wu W-J, Shih C-M. Molecular analysis of Ixodes granulatus, a possible vector tick for *Borrelia burgdorferi sensu lato* in Taiwan. Exp Appl Acarol. 2009;48(4):329–44. doi: 10.1007/s10493-009-9244-4 19184580

[66] Norris DE, Klompen J, Keirans JE, Black IV WC. Population genetics of *Ixodes scapularis* (Acari: Ixodidae) based on mitochondrial 16S and 12S genes. J Med Entomol. 1996;33(1):78–89. doi: 10.1093/jmedent/33.1.78 8906909

[67] Roy BC, Estrada-Peña A, Krücken J, Rehman A, Nijhof AM. Morphological and phylogenetic analyses of Rhipicephalus microplus ticks from Bangladesh, Pakistan and Myanmar. Ticks Tick Borne Dis. 2018;9(5):1069–79. doi: 10.1016/j.ttbdis.2018.03.035 29661691

[68] Kain DE, Sperling FA, Daly HV, Lane RS. Mitochondrial DNA sequence variation in Ixodes pacificus (Acari: Ixodidae). Heredity. 1999;83(4):378–86. doi: 10.1038/sj.hdy.6886110 10583539

