# Correction: A cross-sectional survey of hard ticks and molecular characterization of *Rhipicephalus microplus* parasitizing domestic animals of Khyber Pakhtunkhwa, Pakistan

**DOI:** 10.1371/journal.pone.0307183

**Published:** 2024-07-10

**Authors:** Muhammad Rooman, Yasir Assad, Sadia Tabassum, Samia Sultan, Sultan Ayaz, Muhammad Fiaz Khan, Shahid Niaz Khan, Rehman Ali

The months for seasons Autumn and Summer are incomplete in Table 3. The correct months are “Mar, Apr and May” for Autumn and “Jun, July and Aug” for Summer. Please see the correct Table 3 here.

**Table 3:** Season-wise prevalence of hard ticks in District Bannu.

Variables		Cows	Buffaloes	Sheep	Goats	Overall prevalence%		Pearson’s Chi-square test *(x*^*2*^*)*	*p*-value
Seasons	Months	Infested/ Examined	Infested/ Examined	Infested/ Examined	Infested/ Examined	Month-wise %	Season-wise %		
Spring	Sep	72/83	54/88	18/41	20/41	64.82	65.1	1224.196	0.001
	Oct	43/55	39/64	10/52	12/54	46.22			
	Nov	19/59	27/74	8/79	13/86	22.48			
Winter	Dec	7/56	12/82	4/140	2/143	5.93	11.4		
	Jan	17/60	14/91	4/141	4/141	9.00			
	Feb	29/64	21/65	1/105	7/115	16.61			
Autumn	Mar	46/64	33/64	7/91	7/93	29.80	30.0		
	Apr	64/74	37/71	19/68	8/59	47.05			
	May	68/72	41/60	15/34	13/34	68.50			
Summer	Jun	88/90	59/69	22/40	20/41	78.09	77.5		
	Jul	128/140	71/83	40/56	29/49	83.22			
	Aug	88/89	68/89	38/53	28/44	80.72			
