# Supplementary material for: A cross-sectional survey of hard ticks and molecular characterization of Rhipicephalus microplus parasitizing domestic animals of Khyber Pakhtunkhwa, Pakistan
Source: PLoS One. 2021 Aug 5;16(8):e0255138. doi: 10.1371/journal.pone.0255138 (PMC8341592; doi:10.1371/journal.pone.0255138)
Supplement: S2 Fig — A 2000 ladder was used. 1–3 represent samples of the present study. N represents negative control and P represents the positive control. (DOCX) [file pone.0255138.s002.docx]

**
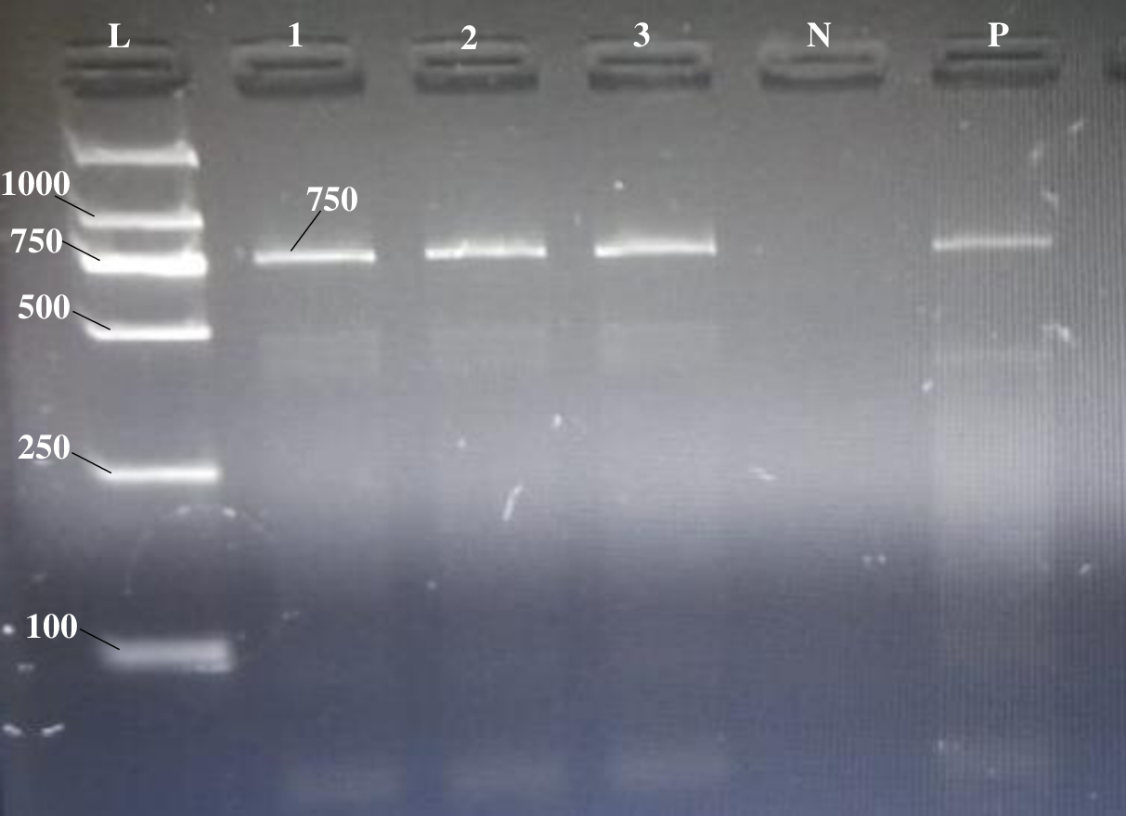
**

**S2 Fig. ITS2 amplified product. A 2000 ladder was used. 1-3 represent samples of the present study. N represents negative control and P represents the positive control**
