# Supplementary material for: A cross-sectional survey of hard ticks and molecular characterization of Rhipicephalus microplus parasitizing domestic animals of Khyber Pakhtunkhwa, Pakistan
Source: PLoS One. 2021 Aug 5;16(8):e0255138. doi: 10.1371/journal.pone.0255138 (PMC8341592; doi:10.1371/journal.pone.0255138)
Supplement: S1 Raw images — (PDF) [file pone.0255138.s003.pdf]

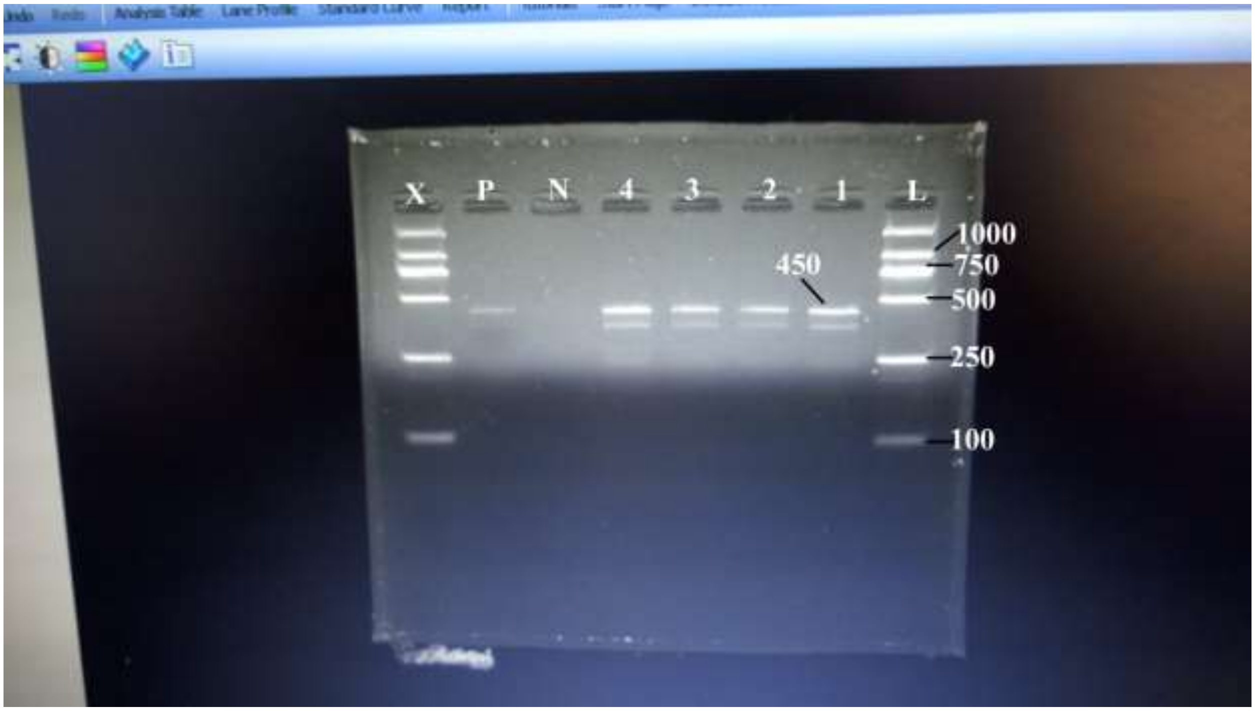

Uncropped Fig. 1: From this uncropped gel, S Fig. 1 of the article was produced. In the S Fig.1 the image was flipped horizontally to make it easy to read.

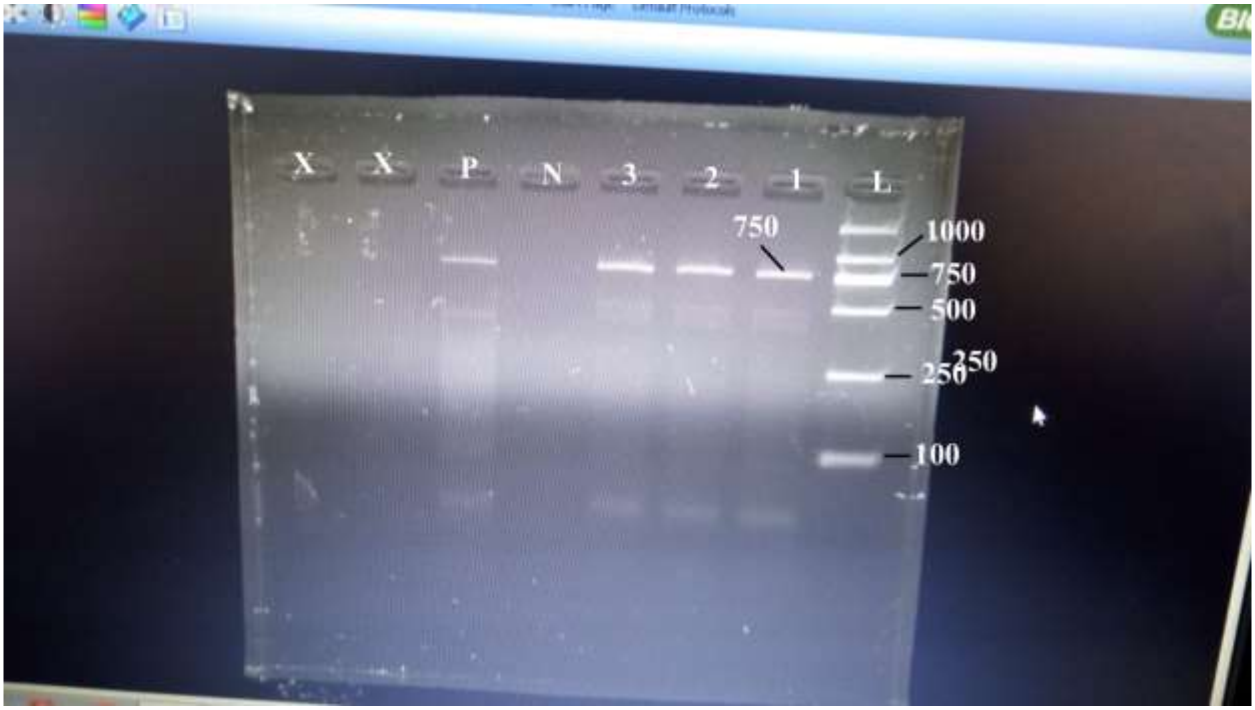

Uncropped Fig. 2: From this uncropped gel, S Fig. 2 of the article was produced. In the S Fig.2 the image was flipped horizontally to make it easy to read.
